# Giving insights into an ICF training: evaluation of an in-person interactive ICF training in Germany

**DOI:** 10.3389/fresc.2024.1419969

**Published:** 2024-07-16

**Authors:** Michaela Kirschneck, Sandra Kus, Michaela Coenen

**Affiliations:** ^1^ Chair of Public Health and Health Services Research, Institute for Medical Information Processing, Biometry, and Epidemiology (IBE), Faculty of Medicine, LMU, Munich, Germany; ^2^ Pettenkofer School of Public Health, Munich, Germany

**Keywords:** functioning, disability, international classification of functioning disability and health, training methodology, ICF training

## Abstract

**Introduction:**

The World Health Organization (WHO) adopted the International Classification of Functioning, Disability and Health (ICF) in 2001. The classification provides a framework for the standardised description of functioning and disability using health and health-related domains. The implementation of the ICF is diverse and has a wide range of applications. A thorough understanding of the ICF classification is essential for successful implementation. We developed and delivered an in-person interactive ICF training to facilitate the implementation of the ICF in Germany. The aim of this paper is to present the evaluation of this in-person interactive ICF training.

**Methods:**

The evaluation was conducted with questionnaires assessing the organisation of the workshops and the knowledge gained during the training using Likert scaled questions. Open-ended questions were used to gather feedback on the further development of the ICF training. Data were analysed descriptively using absolute and relative frequencies. Open-ended questions were analysed qualitatively.

**Results:**

Between 2017 and mid-2020, a team of trainers at the Chair of Public Health and Health Services Research (IBE) at LMU Munich organised 12 in-person interactive ICF trainings with a total of 191 participants. In total 151 participants filled in the questionnaires (response rate: 79.1%). The participants` professional backgrounds were primarily in the social sector (*n* = 76; 50.3%), clinical sector (*n* = 36; 23.8%), and administrative sector (*n* = 31; 20.5%). 42.4% of the participants strongly agreed that the content was relevant to their work, while an additional 51.0% almost agreed. According to this evaluation, 82.1% of the participants would recommend the training to others.

**Discussion:**

A number of constructive suggestions and proposals were made for the further development of the training programme. These mainly related to the content of the training, such as the themes of children and youth and integration assistance.

## 1 Introduction

The World Health Organization (WHO) adopted the International Classification of Functioning, Disability and Health (ICF) in 2001. The classification provides a framework for the standardised description of functioning and disability using health and health-related domains and is, along with the ICD, one of WHO's main classifications (1, 2).

The implementation and use of the ICF is multifarious and covers a wide spectrum of applications, from assessing of functioning of people with disabilities in different settings and different phases of care to the implementation and development of participation programmes. Wherever assessing functioning and disability, all aspects of the integrative biopsychosocial model underlying the ICF classification must be taken into account. The classification comprises over 1,400 alphanumeric codes that categorise and describe information according to the components of the ICF model—body functions and structures, activities and participation, and environmental factors (1). The large number of categories along with its hierarchal coding system as well as the underlying biopsychosocial framework is complex and not self-explanatory (3, 4). Describing health or health-related states of individuals using the ICF therefore requires extensive knowledge of the classification as well as skills in using it as a coding tool (5, 6). Furthermore, the ICF qualifiers enable the description of the extent of the problem in the respective ICF codes. Additionally, with qualifiers, component-specific further characteristics, such as capacity and performance, can be described (1, 7, 8). This knowledge needs to be passed on to users.

Those with a comprehensive understanding of the ICF are well-positioned to facilitate the implementation of international conventions and national laws, such as the Federal Participation Act (BTHG) in Germany. The UN Convention on the Rights of Persons with Disabilities (UN CRPD) was adopted by the United Nations General Assembly in 2006 and relies on the conceptualization and the framework of the ICF as it states the disability as a limitation to participation and as the interaction between a person with a health problem and contextual factors (9). The UN CRPD was the basis for the BTHG, which was passed in Germany in 2017. This legislation created more opportunities for participation and self-determination for people with disabilities. In order to record the respective needs, ICF-based needs assessment tools were developed. It should be noted that the application of these tools requires an understanding of the ICF (10–13).

The need for ICF trainings varies depending on the area of application. This includes both in-person interactive training for users and training for multipliers who will then pass on the knowledge acquired in their respective field of activity or in their institutions, such as early intervention centres, educational institutions or sheltered housing. Different approaches can be used to deliver the ICF related knowledge and skills, such as e-learning tools, virtual training or in-person interactive trainings. We have developed and realised an in-person interactive training for the introduction of the ICF on which we will report in the following.

The general objective of this paper is to present the results of an evaluation of an in-person interactive ICF training. The specific aims are (1) to present the increase in ICF knowledge as a result of the training reported by the participants, (2) to evaluate the training in terms of Intended Learning Objectives (ILOs), content and organisation, and (3) to report on the feedback for further development of the training.

## 2 Methods

With the adoption of the ICF in 2001, a team of researchers at LMU Munich participated in the development of ICF Core Sets projects (14–16) and used the classification in several studies to describe health and health-related states (17–19), respectively. In doing so there was a need to develop and implement an in-person interactive ICF training for research and project partners to facilitate the use of the ICF in these projects. Our experience has demonstrated that it is only possible to attract research and project partners if the theoretical content of the ICF classification and its implementation in the corresponding research project are conveyed, as well as if the participants can gain added value in their daily clinical work and are positively encouraged to implement the classification. In order to achieve this, our concept has been developed on the basis of Klafki's critical and constructive didactics (20). The input came from our empirical experience of the teaching units derived from various research projects. The approach also encompasses multimodal teaching and learning, which entails the utilisation of a diverse array of pedagogical techniques, including frontal teaching with presentations, individual and group work with the presentation of results, and discussion between teachers and learners. This conveys the following active and passive teaching content, which also takes into account the lived experience of the participants (21, 22). The approach, as described by Dewey, is one that increases learning opportunities. This is also taken into account in Kersten Reich's systematic-constructivist didactics, as evidenced in the literature (23).

We developed a one-and-a-half-day training concept based on our expertise with these trainings, to meet the high demand for further trainings for other target groups. The training includes basic knowledge of the biopsychosocial framework and the ICF classification as well as examples of ICF implementation in a broad range of fields.

Since 2017, the team at LMU Munich has implemented an in-person interactive ICF training concept which had been evaluated on a regular basis. The ICF trainings were offered three to five times a year.

### 2.1 Participants

The ICF training was designed for all interested (health) professionals regardless of professional group and field of activity. It is targeted to users, researchers, teachers and trainees with and without previous experience of the ICF.

### 2.2 Instructor team

The training was carried out by members of the LMU Munich, highly experienced in the ICF and ICF trainings. All instructors (MC, MK, SK) are familiar with the ICF classification since its introduction by WHO. The instructors’ experience has been developed through their participation in various ICF research projects, such as the development of ICF Core Sets and the implementation of a large number of studies on the ICF in the context of rehabilitation (14–19, 24, 25).

### 2.3 The in-person interactive ICF training

#### 2.3.1 Advertising and organisation

The training was advertised on LMU website and was also published in the training programme of the Bavarian Medical Association. In addition, participants were made aware of the ICF training programme by word of mouth. The number of participants in an in-person interactive ICF training was limited to 18 to enable interactive group exercises.

#### 2.3.2 Teaching methods

The didactic method is based on the empirical experience of the teaching units derived from various research projects. It incorporates both active and passive teaching content, which also takes into account the lived experience of the participants. The learning units employed various teaching methods, including lectures, individual exercises, and interactive group sessions. The lectures were delivered by the instructors mentioned above on a rotating basis. Individual exercises and interactive group exercises were conducted to deepen the trainees’ knowledge of the ICF. Participants were encouraged to think holistically about potential problems in functioning with a specific health problem, including contextual factors, and were instructed to assign these problems to the different components of the biopsychosocial model. Through the exercises used, the participants familiarised themselves with the structure, the individual ICF codes at the different hierarchical levels as well as the qualifiers. All exercises were supervised by the entire team. Interactive group session exercises were conducted in small groups of 3–5 people. Lecture handouts and exercise materials were provided to participants before each learning unit. Each unit concluded with a brief take-home message.

#### 2.3.3 Intended learning objectives (ILOs) and content of the ICF training

The general intended learning objectives (ILOs) are that participants develop competence in the use of the ICF classification and are able to apply it in everyday professional life, science and teaching using the training documents and ICF classification. The first day of the training provides basic knowledge on the ICF (basic module), while the second day covers in-depth topics. The basic module consists of four learning units: (1) *Introduction to the biopsychosocial model*, ILOs: The participants understand the concept of the ICF and can apply the ICF to case studies (2) *Aim and use of the ICF classification*, ILOs: The participants be able to explain why the description of functioning and disability is important for identifying the needs of individuals and populations as well as functioning and disability is more than a “diagnosis”. (3) *Structure and codes of the ICF classification, ILOs:* The participants can explain how the ICF classification is organised and structured, recognise which of the components a particular ICF Code belongs to and can recognise which hierarchical level of the ICF is reflected in a particular ICF code, and (4) *Qualifiers of the ICF classification*, ILOs: The participants know the qualifiers for evaluating the extent of a problem, know that there are component-specific assessment characteristics and can decode ICF codes with component-specific qualifiers. In unit 1 *Introduction to the biopsychosocial model*, the ICF components are presented and discussed in detail using case studies. Afterwards, the participants work in interactive group sessions to develop a case study on low back pain. Results of the exercise are presented to the group and discussed in detail. In unit 2, *Aim and use of the ICF classification* are presented by showing examples of everyday clinical, social and health care practice. In unit 3, *Structure and codes of the ICF classification,* the hierarchical structure of the classification with its different levels of categories are presented and discussed by a short exercise. In this unit, the lecture is followed by a group session exercise in which health-related information is coded on the basis of case studies (e.g., sections of medical report). The results and any open questions are discussed in plenary. Finally, in unit 4, *Qualifiers of the ICF classification*, the ICF qualifiers are presented and a decoding exercise in a scope of an individual exercise is performed.

**Figure 1:**
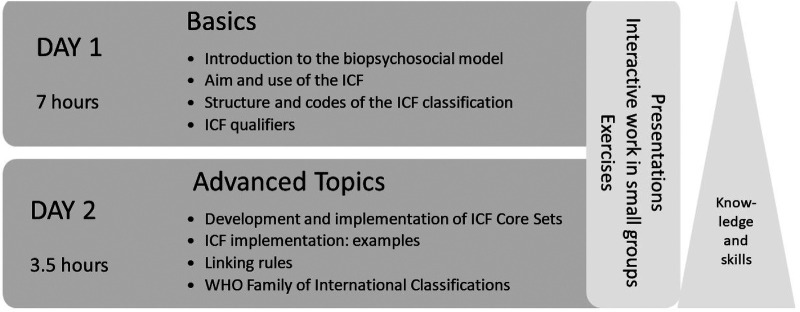
Content of the in-person interactive ICF training.

The second day begins with a brief introductory review of the previous day and then proceeds to demonstrate how the ICF can be utilised in rehabilitation and how documentation can be carried out using an assessment sheet or the functioning profile (7, 26). The development of the ICF Core Sets (27) and their implementation in clinical practice (28) are also presented. Use cases from several institutions in which the ICF as well as the ICF Core Sets had been implemented are described. The ICF linking rules (29–31) for translating health-related information such as reports and questionnaires into the language of the ICF are also presented. This unit includes active individual exercises in which the items of the World Health Organization Disability Assessment Schedule 2.0 (WHODAS 2.0) (32) are translated into the language of the ICF. Finally, the joint use of WHO classifications is presented. The amount of time or content given to the in-depth topics depends on the feedback from the participants during registration (Figure 1).

### 2.4 Evaluation of the training

The evaluation of the ICF training included the following three parts: Documentation of level of ICF knowledge (part 1); assessment of the increase in ICF knowledge (part 2); feedback on the content and organisation of the training as well as documentation of professional background data (part 3). All parts were assessed by using a self-developed and self-administered questionnaire with closed and open-ended questions. A description of the variables is provided in the Appendix.

#### 2.4.1 Documentation of level of ICF knowledge

Participants were asked to indicate their level of knowledge of the ICF using the following response options: (a) no knowledge, (b) basic knowledge, I know the ICF model, (c) I use the ICF in everyday life/at work, (d) I can apply the ICF qualifiers including coding, (e) I am familiar with the linking method.

#### 2.4.2 Assessment of the increase in ICF knowledge

The assessment of the increase in knowledge focused on four topics according to content of the training (first day): (1) the biopsychosocial model, (2) the aim and use of the ICF, (3) the structure and codes of the ICF, and (4) the ICF qualifiers. Increase in ICF knowledge was assessed by means of 17 questions using different formats (e.g., multiple choice questions, fill-in-the-blank and cloze tests). The content of the questions is described by the variables used to evaluate ICF training in the Appendix.

#### 2.4.3 Feedback on the content and organisation of the training/documentation of professional background data

The participants were asked to provide feedback on a number of aspects of the training. These included the organisation of the training, the comprehensibility of the content of the lectures, the appropriateness of the presentations, the improvement in practical application of the ICF as well as the appropriateness of the group exercises, the relevance of the training to the participants’ field of activity, the expectation of acquiring knowledge as a result of the training, and their recommendation of the training. The assessment was conducted using a four-point Likert scale (“strongly agree” to “strongly disagree”). In addition, we asked for comments, remarks or suggestions for improvement regarding the training with an open-ended question. The questionnaire concluded with some basic information on the participants professional background, such as health profession (e.g., physician, nurse, psychologist, social worker, therapist), the field of activity (clinical work, social work, research), as well as years of professional experience.

### 2.5 Data collection

The level of ICF knowledge (part 1) was collected during the registration process. The questionnaire to assess the increase in ICF knowledge (part 2), was completed by the participants after the first day of the training, as it is based on the training content provided on the first day. The questionnaire for obtaining feedback on the content and organisation of the training as well as the professional background data (part 3), were completed following the one-and-a-half-day training. Data collection took place between 2017 and mid-2020 and lasted about 20–30 min.

### 2.6 Data analyses

Data of the closed questions were analysed descriptively using absolute and relative frequencies, as well as mean (M) and standard deviation (SD). The open-ended question was analysed by coding themes. The first author established a category system inductively, which was then checked by the last author. The coding exercise was performed using MAXQDA 22.0 (33).

## 3 Results

Between 2017 and mid-2020, the instructor team organised 12 in-person interactive ICF trainings with a total of 191 participants. On average, 16 participants (range 14–18 participants) attended the training.

According to the information on professional background (part 3)—obtained from 151 participants following the one-and-a-half-day training—the participants` primary fields of professional activity were in the social sector (*n* = 76; 50.3%), followed by the clinical/medical sector (*n* = 36; 23.8%) and the administrative sector (*n* = 31; 20.5%). Further characteristics of the participants are shown in Table 1. Most of the participants had a professional background as social workers (*n* = 50; 33.1%), therapists (*n* = 24; 15.9%), nurses (*n* = 18; 11.9%) and physicians (*n* = 16; 10.6%). The average number of years in the profession was 12.4 years (SD = 8.7).

**Table 1:** Participants’ primary fields of professional activity (*n* = 151).

Participants’ primary fields of professional activity^a^ (*n* = 151)	*n*	(%)
Administration	31	(20.5%)
Social work	76	(50.3%)
Clinical/medical work	36	(23.8%)
Research	19	(12.6%)
Teaching	4	(2.7%)
No information provided	7	(4.6%)

^a^ Multiple responses possible.

### 3.1 Documentation of level of ICF knowledge (part 1)

Upon registration for the course, 71 (37.2%) of the 191 training participants stated that they had no knowledge of the ICF, 48.7% (*n* = 93) reported basic knowledge of the ICF, including knowledge of the biopsychosocial model, 8.4% (*n* = 16) used the ICF in everyday life/at work, 3.1% (*n* = 6) indicated that they had applied the ICF qualifiers, including coding and 0.5% (*n* = 1) reported being familiar with the linking method. Twenty-three participants (12.0%) did not provide information on their ICF knowledge during the registration process (Table 2).

**Table 2:** Level of ICF knowledge (*N* = 191).

Level of ICF knowledge^a^ (*N* = 191)	*n*	(%)
No knowledge	71	(37.2%)
Basic knowledge (ICF model)	93	(48.7%)
Use of ICF in everyday life/at work	16	(8.4%)
Application of ICF qualifiers, including coding	6	(3.1%)
Familiar with the linking method	1	(0.5%)
No information provided	23	(12.0%)

^a^ Multiple responses possible.

### 3.2 Assessment of the increase in ICF knowledge (part 2)

**Figure 2:**
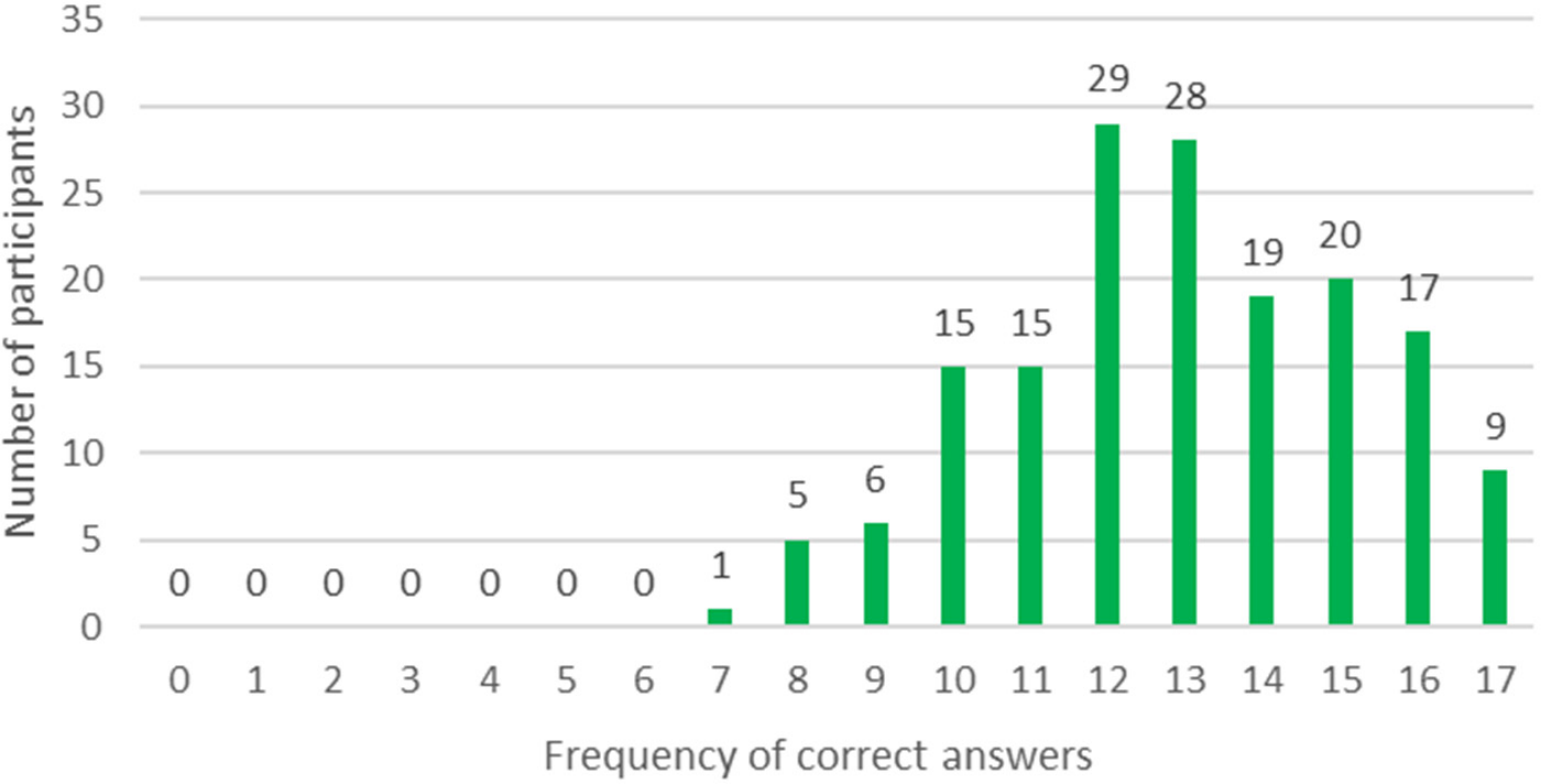
Distribution of the participants’ correct answers (*n* = 164).

We retrieved from a total of 164 participants (85.9% response rate) the questionnaires to evaluate the increase in ICF knowledge. The majority of the 17 questions were answered correctly by the participants (mean 12.9; min–max 7–17). The majority of participants (56.7%, *n* = 39) demonstrated an accurate understanding of the majority of the questions, correctly answering three-quarters or more of the questions. The distribution of the participants’ correct answers is shown in Figure 2. Most questions were answered correctly; in particular, questions on the *Biopsychosocial model* with an average of 82.5% and questions on the *Aim and use of the ICF* with an average of 81.9%. Regarding the four questions on *Structure and codes of the ICF*, it is noticeable that the questions on the structure of the codes and the question on the meaning of the initial letters of an ICF Code (question 4.1 and 4.2; see Appendix) were answered correctly by the majority of the participants (*n* = 144; 87.8% and *n* = 150; 91.5%), while only 112 and 100 participants (68.3% and 61.0%) answered the question on the hierarchical structure of the ICF and the question on the levels of the ICF classification (Question 4.3 and 4.4; see Appendix) correctly. The five questions on the *ICF qualifiers* showed a similar response pattern. Question 5.1 on the basic understanding of the qualifiers (see Appendix) was almost completely answered correctly by all participants (*n* = 160; 97.6%). However, only about half of the participants answered correctly the questions about the meaning and the possible use of component-specific qualifiers (questions 5.3 to 5.5, see Appendix) (*n* = 92; 56.1% to *n* = 78; 47.6%) (see Figure 2).

### 3.3 Feedback on the content and organisation of the training (part 3)

**Figure 3:**
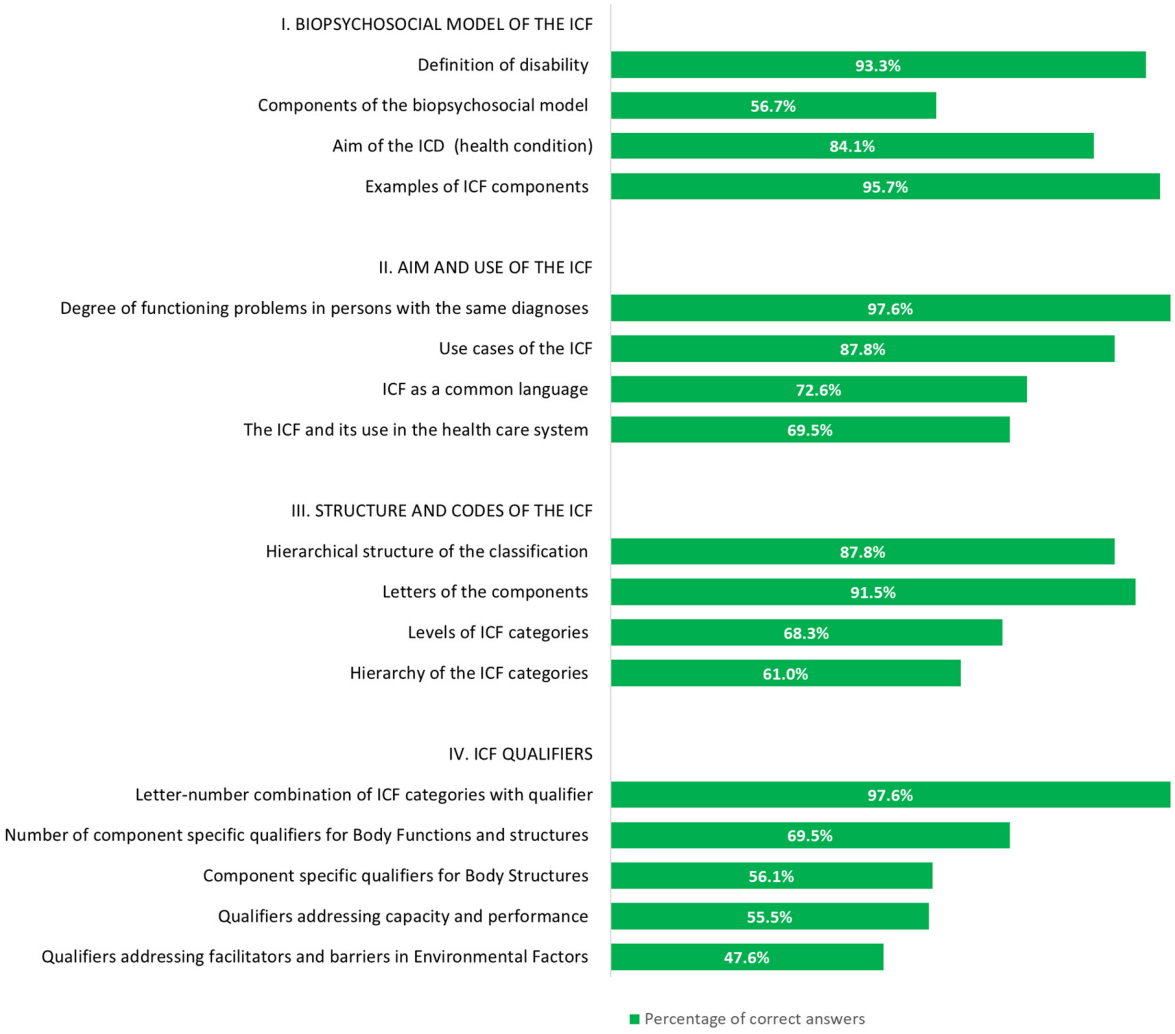
Relative frequencies of correct answers on the questions in knowledge (*n* = 164).

**Figure 4:**
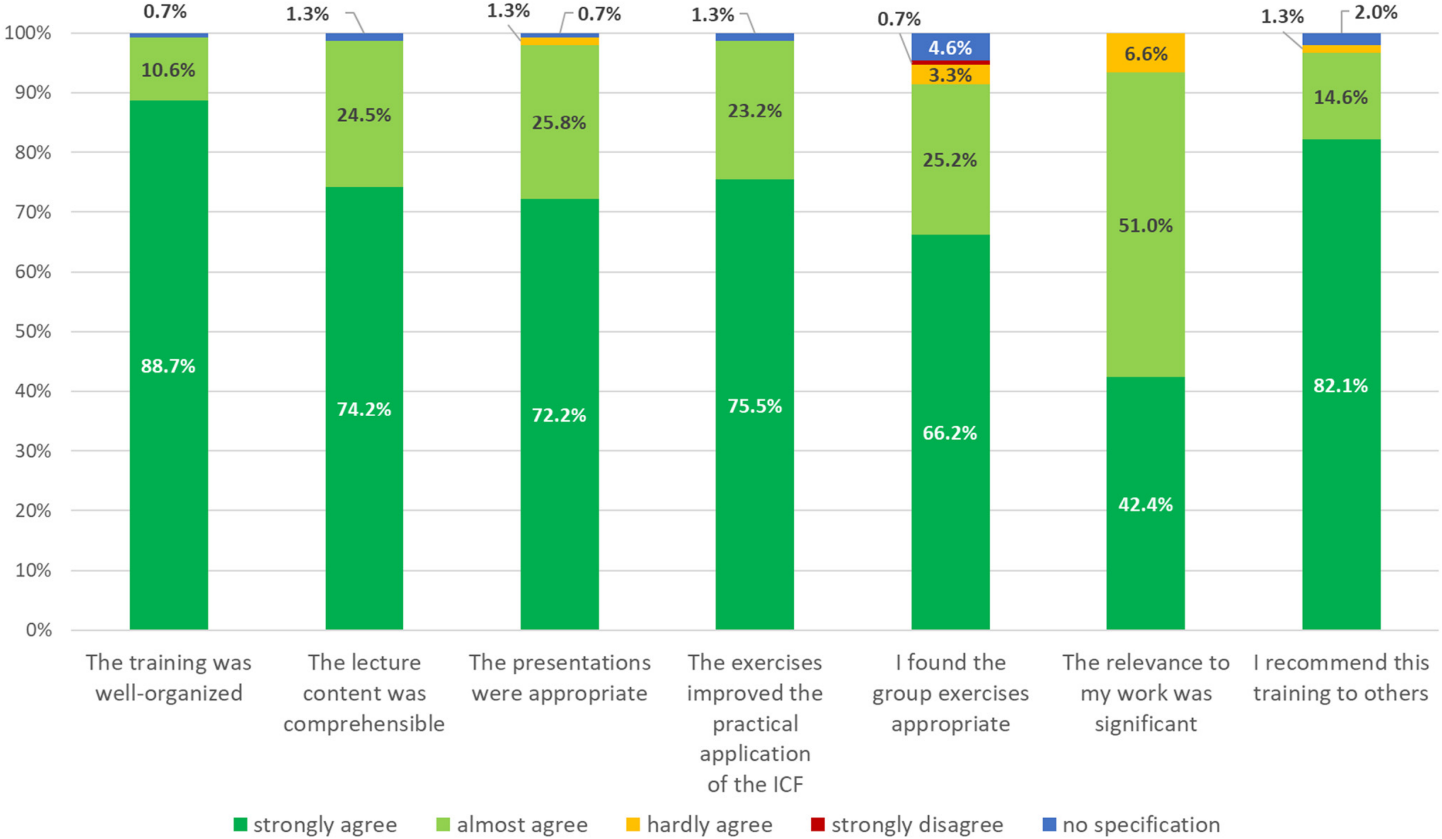
Results of the assessment of the content and organisation of the ICF training (*n* = 151).

We retrieved questionnaires from 151 participants (response rate: 79.1%) to evaluate the content and organisation of the ICF training following the one-and-a-half-day training. The training was mostly rated as well-organized (*n* = 134; 88.7%). It was also reported that the content of the lectures was presented in a comprehensible way (*n* = 112; 74.2%) and the presentations were appropriate (*n* = 109; 72.2%). In total, 75.5%, of the participants (*n* = 114) stated that the exercises improved the skills to apply the ICF in their professional settings and 66.2% (*n* = 100) reported that they found the group exercises appropriate. 64 participants (42.4%) strongly agreed that the training was relevant to their work, while an additional 51.0% (*n* = 77) almost agreed. A total of 124 participants (82.1%) would recommend the training to others (Figure 3) and the majority (98.0%; *n* = 148) of participants strongly or almost agreed that the knowledge acquired during the training met their expectations (Figure 4).

**Figure 5:**
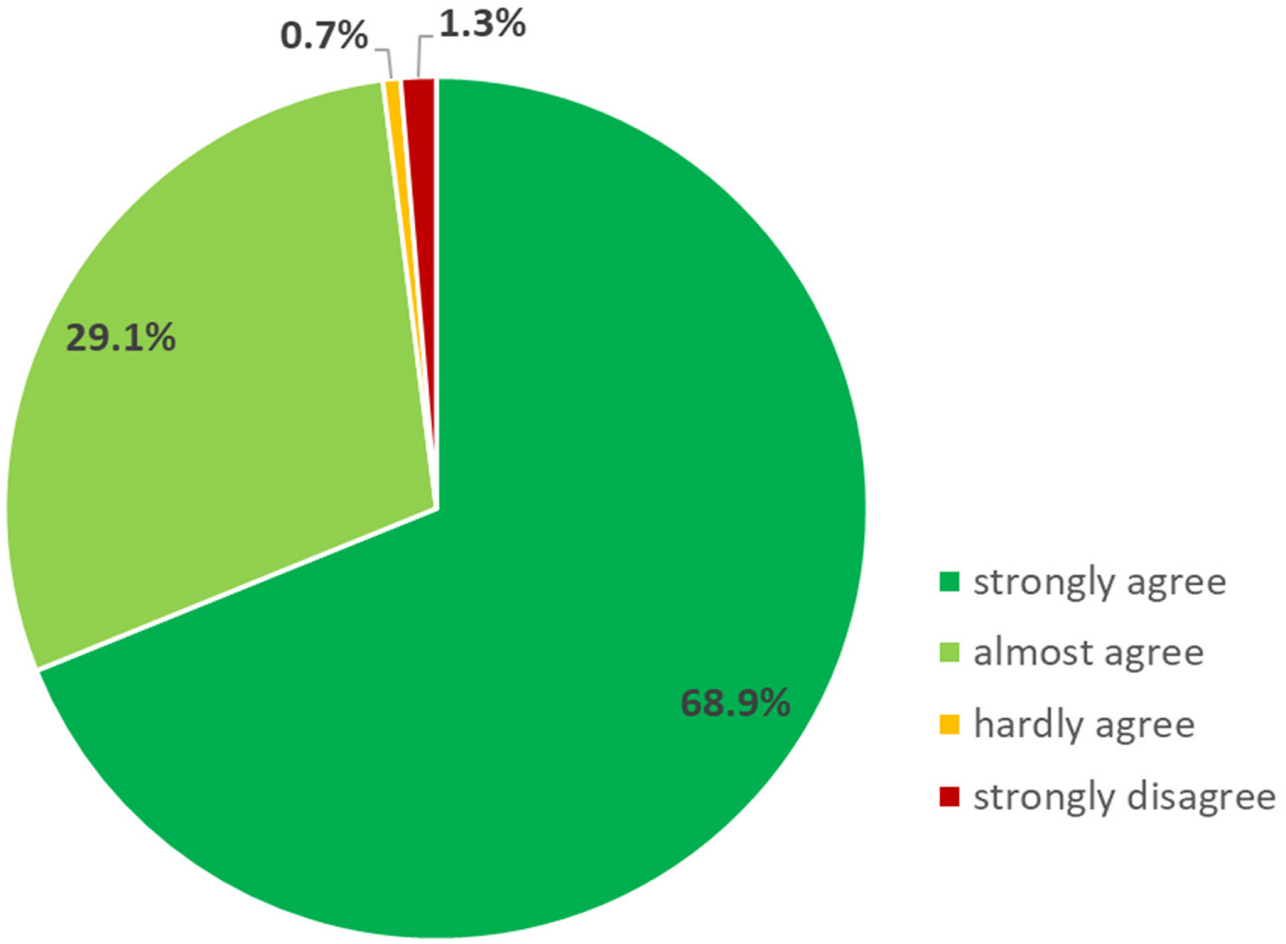
Results on: “the knowledge acquired met my expectations.” (*n* = 151).

In the open-ended questions, 76 participants (50.3%) provided feedback on the training’s content, format and organisation, offering comments and suggestions for improvement. The following overarching themes were retrieved from the answers: *Training in general*, *Organisation of the training*, *Content of the training*, *Participants’ learning objectives*, *Instructors*, *Format of the training*. The number of statements allotted to the themes is shown in Figure 5.

**Figure 6:**
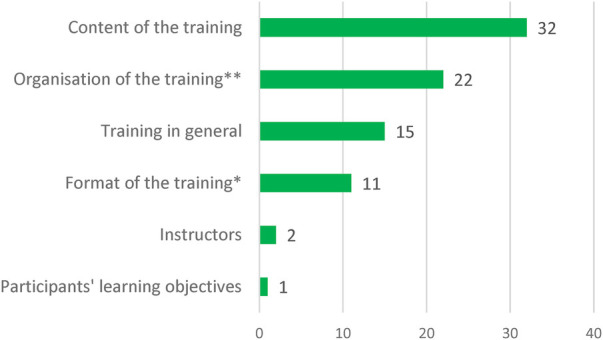
Feedback for further improvement of the training (*n* = 76). Number of statements allocated to the topics with potential improvements (*includes also lectures and presentations; **includes also handouts/scripts, catering).

Most comments and suggestions for improvement were made for the theme *Content of the training*. The need for more information and case studies focusing on children and young people and social support systems was often mentioned, as well as use cases on persons with mental health problems and intellectual disabilities. With regard to another major theme *Organisation of the Training*, it was suggested that the handouts be made available in a more readable format or to make them available digitally. There were also suggestions for ICF networking, requests to be informed about further training opportunities and suggestions to carry out the assessment of learning objectives on the second day. Suggestions coded to the theme *Training in general* were mainly related to the length of the training. It was suggested that the training should be split into two full days, as the first day (basic module) was felt to be very compact and demanding in terms of content. However, there was also need for more time for discussion and for the second day to be more in-depth. Another theme was the *Format of the training*. There were requests for more specific tasks in the group exercises and an adapted exercise time. The take-home massage on the last slide of each lecture was mentioned as helpful (Figure 6).

## 4 Discussion

This paper reports on the evaluation of a one-and-a-half day in-person interactive ICF Training to facilitate the implementation of the ICF in Germany. This ICF training was carried out 12 times between 2017 and mid-2020, with 191 participants. The evaluation showed a high level of satisfaction with the content and organisation of the training offered at the LMU Munich. Most participants described the content as relevant for their field of work and the increase in knowledge met the participants` expectations. A number of constructive suggestions were made for the further development of the training, mainly related to the content of the training. Overall, the majority of participants were very or mostly satisfied with the ICF training and would recommend it to others.

In general, training courses are well received if the participants benefit from them in their professional or educational work (34). The relevance of a training course to professional activities and tasks is a prerequisite for a high level of satisfaction with a training. Almost all of our participants confirmed this regarding the ICF training they had attended. In addition, a varied and interactive didactic design is favourable for grasping new topics (35, 36). This ICF training is jointly conducted by three experienced instructors who have been researching and teaching ICF topics since the ICF classification was first adopted. The theoretical content is taught in lectures given by a single instructor. Through the interactive group sessions and individual exercises, supervised by the entire team of instructors, the participants were able to contribute and implement their own lived experience (37). Open questions were addressed in all teaching units. This approach was very well received by the participants, who reported that they enjoyed the group work and found the presentations very appealing and easy to understand. The ability to successfully apply what has been learnt, for example in group work, is motivating and can give confidence to transfer it to a professional context. In addition, open questions arising during the exercises can be directly addressed and answered by the supervising instructors.

The development of skills and competencies, which are promoted through interactive group and individual exercises, can lead to success and confidence and may be linked to participant satisfaction. Suggestions for improving the content of the ICF training were mainly related to the desire for more time for discussion and exercises, as well as the inclusion of more specific application areas and examples from the different contexts of the participants. The participants` professional background was very heterogeneous, which stimulated discussions from different perspectives. These discussions highlighted the wide range of applications of the ICF. Konstanjesk et al. (2011) describe the numerous potential applications of the ICF. They discuss its application as a conceptual framework, as well as the terminology and coding capability of the ICF in different fields for instance health and disability data collection in surveys of general and specific populations, development of disability survey modules & question sets or policy development and monitoring (38). In doing so, they emphasise the fundamental importance of a common language within the ICF. The discussion with the participants also made it clear that the application of the ICF classification varies. For example, coding with its qualifiers is not yet used in some areas, and the ICF is initially only used as a conceptual framework with its ICF language. It can be observed that the requisite knowledge and skills for ICF applications vary according to the specific context. Furthermore, the heterogeneous participants engaged in the discussions demonstrated that the ICF offers the potential and benefits for multidisciplinary and intersectional collaboration (4, 39).

The in-person interactive ICF training concept includes many examples and exercises, ranging from rehabilitation planning and documentation (7, 40) to scientific applications and the implementation of the UN Convention on the Rights of Persons with Disabilities (UN CRPD) (12, 13, 41). However, some professional groups have requested more examples from their specific fields, such as early intervention and youth services, addiction and related conditions, including those of people with multiple disabilities, especially in the area of mental health. A potential issue arises due to the fact that, on the one hand, it is important to have a diverse range of professionals in the training course to facilitate mutual learning; on the other hand, however, it is also crucial to consider the specific interests and individual needs of the participants in their respective fields of application. The multidisciplinary and intersectional approach, the philosophy of the ICF and the positive feedback on the current training support the need for further consideration.

From the participants’ point of view, the increase of knowledge on the ICF is predominantly high and very satisfactory. In particular, the objectives and benefits of the ICF and the content of the biopsychosocial model were particularly well implemented and accepted, which is probably due to the political and social requirements and thus the increased interest of the participants. With the adoption of the UN Convention on the Rights of Persons with Disabilities (UN CRPD) on 13 December 2006, the biopsychosocial model is of particular importance. The UN CRPD adopts the terminology of the ICF by considering disability as a limitation to participation, resulting from the interaction between an individual’s health condition and their contextual factors. It reaffirms and concretises universal human rights in relation to the situation of people with disabilities and thus forms the basis for the equal, full and effective participation of people with disabilities in political, social, economic and cultural life (9). Accordingly, basic knowledge of the classification is necessary for the implementation of the requirements of the UN CRPD for all actors who are active in the reporting and application process for persons with disabilities or who work with them. To meet this need, training courses need to be made available to all ICF users to enhance their knowledge of the ICF.

The results of the evaluation reveal that the hierarchical structure of the ICF with its different levels, as well as the component-specific qualifiers could not be achieved by all participants. This demonstrates the complex nature of the ICF classification (3) and its wide scope of applications (41, 42). Some participants may need more time to practice in order to consolidate this content. Delivering the content in other formats may also be helpful. In addition to extending the time spent on interactive group and individual exercises, which have been shown to be beneficial, it may be necessary to present difficult content several times and to look at it from different perspectives. It may also be helpful for some participants to prepare this content in small steps. Few participants expressed the wish for context-specific in-depth knowledge on the second day and for basic knowledge to be spread over several days.

Extending the training from 1.5 to 2 days could provide even more time for exercises and discussions. In addition to the didactic concept, good organisation of the training is also important for the participants. This involves managing participants by providing them with advance information about the event and communicating with them, as well as ensuring that the general conditions are met. A pleasant atmosphere is determined by the quality of the available rooms and catering and is closely related to the available resources. The training took place in a seminar room at LMU Munich, where up to 20 people can be trained. The organisation of the training was predominantly rated as very satisfactory. It can therefore be assumed that the framework conditions, such as the group size, also positively contributed to satisfaction.

A detailed and well-founded knowledge of the ICF is essential for all actors to be able to transfer it to the specific fields. In-depth knowledge particularly in the structure and codes of the ICF, which is explicitly practised in the in-person interactive training through coding and decoding units, is of paramount importance. It enables participants to acquire coding skills and competencies, which in turn allows them to code their ICF application context into the language of the ICF. This is relevant in research and teaching, as well as in areas where documents, reports or questionnaires are translated into the language of the ICF, or where ICF-based assessment instruments are used. The current era of digitalisation and the associated development of various ICF tools, such as the ICF coding tool and other tools for ICF integration in electronic health and rehabilitation record systems marks a significant shift from manual to digital documentation and coding with ICF. A position paper “Framework for the WHO-FIC Network Strategic Plan 2021–2026” outlining the strategic coordination has been published on the WHO Family of International Classifications (WHO-FIC) website since February 2024 (43). An adequate familiarity with the ICF will continue to be essential for the effective utilisation of numerous tools and the creation of further tools. The introduction of new digital ICF application formats and digital ICF tools will necessitate further training in ICF, with particular emphasis on the digitalisation aspect. This has to be considered in the future development of our training content. Furthermore, this fundamental understanding also facilitates comprehension of the other classifications within the WHO-FIC, such as the ICD-11 (2) or ICHI (International Classification of Health Interventions) (44), in which the coding scheme is also analogous. This ICF knowledge is also currently relevant in Germany in the context of the needs assessment for people with disabilities. Since 2017, all federal states are obliged to develop and implement ICF-based assessment instruments (10). This need for specific ICF knowledge is also reflected in the sectors in which the participants work. More than half of the training participants came from the social sector (social work), presumably working mainly in the context of integration assistance and needs assessment. One approach to meet the specific needs of participants would be to offer in-depth and specific in-person ICF training focusing on the needs assessment implemented in Germany. The focus should be on the higher hierarchical structure of the classification; the component specific qualifiers, especially in the components activities and participation as well as environmental factor, should be practised more intensively. The component-specific qualifiers in the environmental factors describe the environment as either a facilitator or a barrier and relate to a person’s activity and participation. The component-specific qualifiers of the activity and participation component are described under two different aspects. The first aspect concerns performance. Here, the impact of environmental factors is reflected. The second aspect concerns capacity. In this context, the individual is described in a uniform, standard environment, without assistance. This description enables the mapping of the needs of people with disabilities for optimal participation, which can then be addressed within the scope of integration assistance as well as early intervention.

Another way of consolidating what had been learnt could be to repeat what has been learnt by going through the handouts again in self-study or by using them as a reference book. Our results show that this option could be relevant for the participants. In addition, the ICF eLearning tool could also be used to review the learning. In collaboration with the WHO-FIC, an ICF eLearning tool has been developed to provide a standardised learning programme for all interested participants worldwide. It consists of several modules, suitable for different areas of application: Clinical Practice, Health and Disability Statistics, Disability and Social Services, Research and Education (30). The tool is currently available in ten languages, with three more (German, Japanese and Korean) to be added soon (45, 46).

It is crucial to emphasise that the collection of data is absolutely anonymous. All three parts, namely the *Documentation of level of ICF knowledge*, *Assessment of the increase in ICF knowledge* and *Feedback on the content and organisation of the training* are recorded separately and independently from each other. Consequently, no data allocation can be performed and the interference statistics will be limited as a result.

## 5 Conclusion

The one-and-a-half-day in-person interactive ICF training programme has been successfully implemented in Germany. The evaluation showed that a modification of the training should be considered in order to meet the needs of the participants. With the implementation of the UN CRPD, the need for comprehensive training will remain high. In Germany, implementation is enshrined in the BTHG, which includes a nationwide needs assessment using ICF-based tools. Successful implementation of needs assessment depends, among other things, on the ability of users to understand and apply the concept and structure of the ICF. The know-how acquired in the ICF training paves the way for individual application by all those trained. The focus of the in-person interactive ICF training is to create a basis for the application and implementation of the ICF classification that can be transferred to a wide range of uses. The introduction of new digital ICF application formats and digital ICF tools will necessitate further training in ICF, with particular emphasis on the digitalisation aspect.

## Data Availability

The raw data supporting the conclusions of this article will be made available by the authors, without undue reservation.
