# Supplementary material for: Giving insights into an ICF training: evaluation of an in-person interactive ICF training in Germany
Source: Front Rehabil Sci. 2024 Jul 16;5:1419969. doi: 10.3389/fresc.2024.1419969 (PMC11286556; doi:10.3389/fresc.2024.1419969)
Supplement: Supplementary file 1 [file Datasheet1.pdf]

Variables used for the evaluation of the ICF training

Format of response options is presented in brackets.

Part 1 Documentation of level of ICF knowledge

1.1 Knowledge of the ICF (no knowledge, basic knowledge, I know the ICF model, I use the ICF in everyday life/at work, I can apply the ICF qualifiers including coding, I am familiar with the linking method)

Part 2 Assessment of the increase in ICF knowledge

(A) Biopsychosocial model

2.1 Definition of disability (yes – no)
2.2 Components of the biopsychosocial model (multiple choice with 5 response options)
2.3 Aim of the ICD (yes – no)
2.4 Examples of ICF components (sorting task with 5 pairs)

(B) Aim and use of the ICF

3.1 Degree of functioning problems in persons with the same diagnoses (yes – no)
3.2 Use cases of the ICF (multiple choice with 5 response options)
3.3 ICF as a common language (cloze test)
3.4 The ICF and its use in the health care system (multiple choice with 5 response options)

(C) Structure and codes of the ICF

4.1 Hierarchical structure of the classification (yes – no)
4.2 Letters of the components (sorting task with 4 pairs)
4.3 Levels of ICF categories (multiple choice with 4 response options)
4.4 Hierarchy of the ICF categories (yes – no)

(D) ICF qualifiers

5.1 Letter-number combination of ICF categories (yes – no)
5.2 Number of component specific qualifiers for Body Functions and structures (yes – no)
5.3 Component specific qualifiers for Body Structures (sorting task with 3 pairs)
5.4 Qualifiers addressing capacity and performance (multiple choice with 5 response options)
5.5 Qualifiers addressing facilitators and barriers in Environmental Factors (multiple choice with 4 response options)

Part 3 Feedback on the content and organisation of the training / documentation of professional background data

6

6.1 The training was well-organized (4-point Likert scale)
6.2 The lecture content was comprehensible (4-point Likert scale)
6.3 The presentations were appropriate (4-point Likert scale)
6.4 The exercises improved the practical application of the ICF (4-point Likert scale)
6.5 I found the group exercises appropriate (4-point Likert scale)
6.6 The relevance to my work was significant (4-point Likert scale)
6.7 The knowledge acquired met my expectations (4-point Likert scale)
6.8 I recommend this training to others (4-point Likert scale)
6.9 Comments, remarks or suggestions for improvement regarding the training (Open-ended question)

6.10 Health profession (physician; nurse; psychologist, social worker, therapist, others)
6.11 Field of activity (clinical work, social work, research, others)
6.12 Year of professional experience
